# Comparison of the effects of albumin and crystalloid on mortality in adult patients with severe sepsis and septic shock: a meta-analysis of randomized clinical trials

**DOI:** 10.1186/s13054-014-0702-y

**Published:** 2014-12-15

**Authors:** Jing-Yuan Xu, Qi-Hong Chen, Jian-Feng Xie, Chun Pan, Song-Qiao Liu, Li-Wei Huang, Cong-Shan Yang, Ling Liu, Ying-Zi Huang, Feng-Mei Guo, Yi Yang, Hai-Bo Qiu

**Affiliations:** Department of Critical Care Medicine, Zhongda Hospital, School of Medicine, Southeast University, 87 Dingjiaqiao Rd, Nanjing, 210009 P.R. China

## Abstract

**Introduction:**

The aim of this study was to examine whether albumin reduced mortality when employed for the resuscitation of adult patients with severe sepsis and septic shock compared with crystalloid by meta-analysis.

**Methods:**

We searched for and gathered data from MEDLINE, Elsevier, Cochrane Central Register of Controlled Trials and Web of Science databases. Studies were eligible if they compared the effects of albumin versus crystalloid therapy on mortality in adult patients with severe sepsis and septic shock. Two reviewers extracted data independently. Disagreements were resolved by discussion with other two reviewers until a consensus was achieved. Data including mortality, sample size of the patients with severe sepsis, sample size of the patients with septic shock and resuscitation endpoints were extracted. Data were analyzed by the methods recommended by the Cochrane Collaboration Review Manager 4.2 software.

**Results:**

A total of 5,534 records were identified through the initial search. Five studies compared albumin with crystalloid. In total, 3,658 severe sepsis and 2,180 septic shock patients were included in the meta-analysis. The heterogeneity was determined to be non-significant (*P* = 0.86, I^2^ = 0%). Compared with crystalloid, a trend toward reduced 90-day mortality was observed in severe sepsis patients resuscitated with albumin (odds ratio (OR) 0.88; 95% CI, 0.76 to 1.01; *P* = 0.08). However, the use of albumin for resuscitation significantly decreased 90-day mortality in septic shock patients (OR 0.81; 95% CI, 0.67 to 0.97; *P* = 0.03). Compared with saline, the use of albumin for resuscitation slightly improved outcome in severe sepsis patients (OR 0.81; 95% CI, 0.64 to 1.08; *P* = 0.09).

**Conclusions:**

In this meta-analysis, a trend toward reduced 90-day mortality was observed in severe sepsis patients resuscitated with albumin compared with crystalloid and saline. Moreover, the 90-day mortality of patients with septic shock decreased significantly.

**Electronic supplementary material:**

The online version of this article (doi:10.1186/s13054-014-0702-y) contains supplementary material, which is available to authorized users.

## Introduction

Severe sepsis and septic shock are major causes of death in critically ill patients, and fluid management can resuscitate patients in these conditions. Early adequate volume expansion is emphasized as the key elements to be focused on saving lives [1-3]. With the advantage of restoring effective volume and maintaining colloid osmotic pressure, albumin is considered to be administered in addition to crystalloid in the initial fluid management of patients with severe sepsis and septic shock [4].

Whether albumin is associated with reduced mortality in the resuscitation of severe sepsis and septic shock compared with crystalloid is a matter of debate. The predefined subgroup analysis of the Saline versus Albumin Fluid Evaluation (SAFE) study suggested albumin potentially resulted in lower mortality in patients with severe sepsis [5,6]. However, previous meta-analyses that focused on sepsis patients drew nonequivalent results [7,8]. The large randomized controlled trial, the Albumin Italian Outcome Sepsis (ALBIOS) study also reported contradictory results that albumin provided no improvement at day 28 in patients with severe sepsis [9], while the *post hoc* analysis of the septic shock subgroup supported a survival benefit to albumin, given the target patient population in this meta-analysis.

As a result of few investigators emphasizing the use of albumin in patients with severe sepsis, let alone septic shock, a meta-analysis is needed to address the outcome of albumin as fluid therapy. Furthermore, as the most commonly used crystalloid, no study stressed the role of albumin when compared with saline in severe sepsis patients.

Our goal was to examine whether albumin reduced mortality when employed in the resuscitation of adult patients with severe sepsis and septic shock compared with crystalloid solutions and saline.

## Material and methods

### Search strategy for identification of relevant studies

A search of the following databases was conducted: Medline, Elsevier, Cochrane Central Register of Controlled Trials and Web of Science databases. The following keywords were used as searching terms: ‘albumin’ or ‘serum albumin’ or ‘albumin replacement’ or ‘colloid’ or ‘crystalloid’ or ‘crystalloid solution’ or ‘saline’ or ‘normal saline’ or ‘Ringer's solution’ or ‘bicarbonated Ringer’s solution’ or ‘lactated Ringer’s solution’ or ‘isotonic solution’ or ‘hypertonic saline’ and ‘sepsis’ or ‘severe sepsis’ or ‘septic shock’ or ‘shock’ or ‘critical ill’ or ‘critical illness’ or ‘intensive care units’ or ‘intensive care’ or ‘critical care’ or ‘ICU’. No language restrictions were placed on the search. All databases were searched for articles published from inception until March 31, 2014. Additional files and supplementary appendices of the relevant articles were also reviewed. Detailed search strategies are shown in Additional file 1: Search strategy.

### Study selection

One reviewer screened the search results, and the full-text manuscripts of all potentially eligible studies were acquired. Then, all of the articles were reviewed by two reviewers independently in accordance with the inclusion criteria. Disagreements between the two reviewers were resolved by consensus and discussion including a third reviewer. When the ‘same author’ or ‘same data’ issue was confronted with, the latest published study was included.

### Inclusion and exclusion criteria

We included trials with the following features:

1. Type of trials: randomized controlled clinical and parallel trials.
2. Population: trials including adult population with severe sepsis. Severe sepsis was defined as sepsis plus sepsis-induced organ dysfunction or tissue hypoperfusion [4].
3. Intervention: patients submitted to albumin for fluid therapy.
4. Comparison: crystalloid for fluid therapy.
5. Outcome: the primary outcome was all-cause mortality, including 28-day mortality, 90-day mortality or mortality at other time points.

Trials with the following features were excluded:

1. They were not published in English or Chinese.
2. They were not published as original articles.
3. They did not use adult patients.
4. They did not compare albumin with crystalloid.
5. They included no data on mortality in patients with severe sepsis.
6. Full-text articles were not available.

### Quality assessment

The quality of each article was assessed by two reviewers independently. Disagreements were resolved by consulting a third reviewer. The five-point Jadad scale was calculated to assess the quality of the trial [10]. This scale includes the method of randomization, blinding, and loss to follow-up. In addition, sequences generation, allocation concealment, incomplete outcome data, selective reporting and other bias were also inspected to assess the risk of bias [11]. The latter was reported as low risk, unclear risk, or high risk for each trial. Low risk was defined as low risk of bias in all domains. Unclear risk was defined as unclear risk of bias in at least one domain with no high risk of bias domains. High risk was defined as high risk of bias in one or more domains. The publication bias was assessed by funnel plot techniques.

### Data extraction and management

Using a data extraction table, two reviewers independently extracted data. Disagreements were resolved by discussion with other two reviewers until a consensus was achieved. Then, data were proofread by another reviewer. Mortality data, including 28-day or 90-day mortality, were recorded during the data extraction. When 28-day and 90-day morality values were both published in the article, the longest complete follow-up was preferentially used for evaluation in the meta-analysis. However, when 28-day or 90-day mortality values were not presented, ICU or hospital mortality or mortality at other time points were recorded. Other data including the population of each trial, sample size of the trial and patients with severe sepsis, and resuscitation endpoints were extracted. If there was insufficient information in the publications, the contact to the authors was performed.

For studies including a subgroup of septic shock patients, the sample size of septic shock patients and the number of non-survivals were extracted.

### Statistical analysis

Data were analyzed by Review Manager 4.2 (The Nordic Cochrane Center, Rigshospitalet, Copenhagen, Denmark). The pooled odds ratio (OR) for dichotomous data and mean differences for continuous data with 95% confidence intervals (CIs) were calculated. The statistical heterogeneity of the data was explored and quantified using the Mantel-Haenszel chi-square test and the I^2^ test. Heterogeneity was predefined as *P* <0.05 with the Mantel-Haenszel chi-square test or an I^2^ value >50%. The random-effects model was used if heterogeneity was observed; otherwise, the fixed-effects model was used. *P* <0.05 was considered statistically significant.

## Results

### Study location and selection

A total of 5,534 records were identified through the initial search, and 2,330 records were removed as duplicates. The remainder of the 3,204 records was screened. After assessment of the titles and abstracts, 2,637 articles were excluded as not relevant, and 519 articles were excluded for including pediatric patients. In total, 48 potentially eligible studies were identified for inclusion. Six studies [6,9,12-15] compared albumin with crystalloid solutions. Of the included trials, two trials [12,13] were performed and published by the same group in adjacent years. The above factors as well as the similar sample size and mortality rates provided reasonable doubt that the enrolled patients might be the same, and the ‘same author’ or ‘same data’ issue was confronted. Therefore, the latest published study was included, and the trial by Haupt *et al*. [12] was excluded. Finally, 3,658 severe sepsis and 2,180 septic shock patients were included in the meta-analysis.

**Figure 1:**
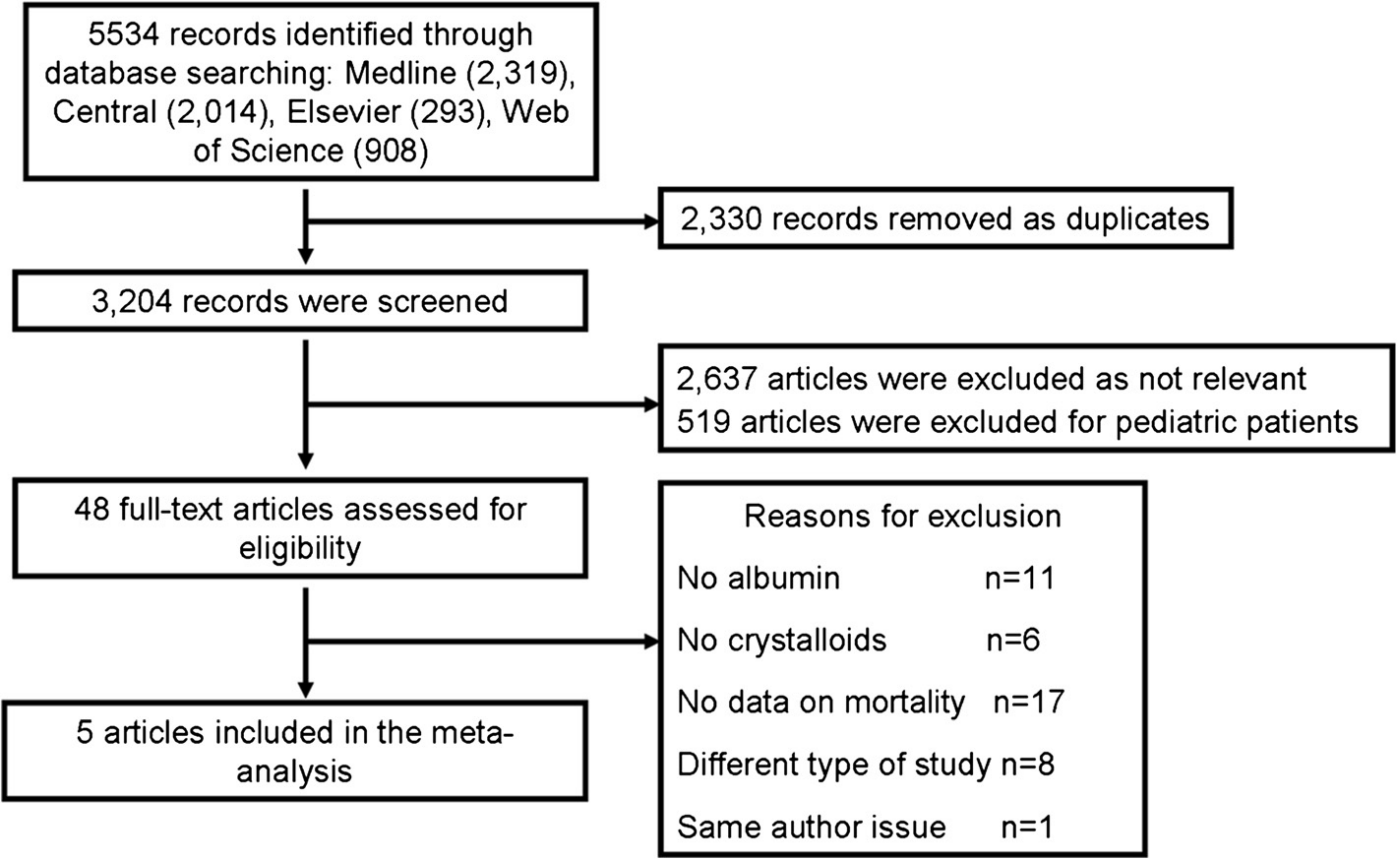
**Flow diagram of the search process and study selection.**

Detailed excluded articles are shown in Additional file 2: Excluded articles. The flow diagram is presented in Figure 1.

### Study characteristics

The characteristics of the included studies are shown in Table S1 in Additional file 3. In total, 4 or 5% albumin was used for fluid resuscitation in three trials [6,13,15], and 20% albumin was employed for volume expansion in one trial [9]. The remaining one [14] included both concentrations. Normal saline was used as crystalloid in two trials [6,13], and Ringer’s lactate solution was used as crystalloid in one trial [15]. The remaining two trials [9,14] included a broad variety of crystalloid products. Mortality was explored for more than one time point, including 28-day mortality and 90-day mortality rates, in two articles, the CRISTAL study [14] and the ALBIOS study [9]. Only 28-day mortality values were published in the SAFE study [6]. The hospital mortality was published in the remaining two trials [13,15]. Quality assessments of the included studies are shown in Table 1.

**Table 1:** **Quality assessment of the included studies**

**Authors**	**Sequences generation**	**Allocation concealment**	**Blinding of participants and researchers**	**Blinding of outcome assessment**	**Incomplete outcome data**	**Selective reporting**	**Other bias**	**Overall risk of bias**
Rackow *et al*. [13]	Unclear	High	High	High	Low	Unclear	Low	High
Metildi *et al*. [15]	Low	High	High	High	Low	Unclear	Low	High
The SAFE study investigators [6]	Low	Low	Low	Low	Low	Unclear	Low	Unclear
The CRISTAL study investigators [14]	Low	Low	High	Low	Low	Unclear	Low	High
The ALBIOS study investigators [9]	Low	Low	High	High	Low	Low	Low	High

Sequence generation: Rackow *et al.* [13] used predetermined randomization schedule. Metildi *et al*. [15] used random numbers. SAFE [6] used a central minimization algorithm. CRISTAL [14] and ALBIOS [9] used computer-generated sequences. Allocation concealment: no detailed allocation concealment was reported by Metildi *et al*. [15]. Rackow *et al.* [13] used a predetermined randomization schedule. SAFE [6] used a central minimization algorithm. CRISTAL [14] used sealed envelopes. ALBIOS [9] used a blinded assignment sequence. Blinding: blinding was not reported in Rackow *et al.* [13], Metildi *et al*. [15] and the ALBIOS [9] trial. Blinding was performed in the SAFE [6] trial. Data collection members but not researchers were blinded in the CRISTAL [14] trial. Incomplete outcome data: all trials described the follow-up. Selective reporting: the study protocol of ALBIOS [9] was obtained. Other bias: no evidence of other sources of bias.

### The impact on mortality in severe sepsis patients

**Figure 2:**
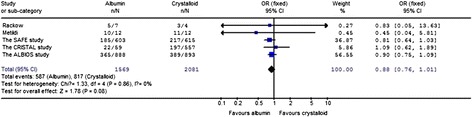
**The effect of albumin on 90-day mortality in patients with severe sepsis.**

The effects of resuscitation with albumin on 90-day mortality in patients with severe sepsis was estimated from five trials (Figure 2), and the heterogeneity was also determined to be non-significant (*P* = 0.86, I^2^ = 0%). The longest complete follow-up mortality rates of all of the trials were evaluated in the meta-analysis. In two articles [9,14], mortality rates were explored for more than one time point, and 90-day mortality rates were analyzed for these two trials. A trend toward reduced 90-day mortality was observed in severe sepsis patients resuscitated with albumin compared with crystalloid. However, the finding was not statistically significant (OR 0.88; 95% CI, 0.76 to 1.01; *P* = 0.08).

**Figure 3:**
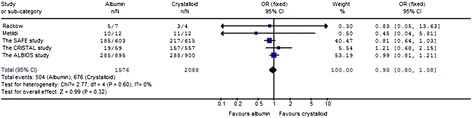
**The effect of albumin on 28-day and hospital mortality in patients with severe sepsis.**

The 28-day mortality was also evaluated (Figure 3). Compared with crystalloid, albumin displayed no beneficial effect on 28-day mortality in severe sepsis patients (OR 0.93; 95% CI, 0.80 to 1.08; *P* = 0.32).

### The impact on 90-day mortality in patients with septic shock

**Figure 4:**
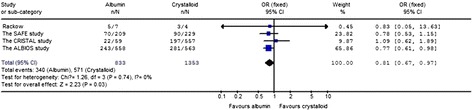
**The effect of albumin on 90-day mortality in patients with septic shock.**

The impact of albumin on 90-day mortality in patients with septic shock was estimated from five trials (Figure 4), the heterogeneity was determined to be non-significant (*P* = 0.74, I^2^ = 0%). The longest complete follow-up mortality rates of all the trials were evaluated, including the 90-day mortality values from the CRISTAL study [14] and the ALBIOS study [9], 28-day mortality values from the SAFE study [6] and hospital mortality from the Rackow *et al.* [13] studies. Compared with crystalloid, the use of albumin for resuscitation significantly decreased the 90-day mortality in septic shock patients (OR 0.81; 95% CI, 0.67 to 0.97; *P* = 0.03). The studies of septic shock patients included in the meta-analysis are shown in Table 2.

**Table 2:** **Patients with septic shock included in the meta-analysis**

**Authors**	**Year**	**Definition of septic shock**	**Sample size of septic shock**	**Non-survival of septic shock**	**Non-survival of albumin group with septic shock (events/total)**	**Non-survival of crystalloid group with septic shock (events/total)**
Rackow *et al*. [13]	1983	Patients were considered septic shock if (1) blood cultures were positive and/or an infected focus was identified, (2) systolic intra-arterial pressure of less than 90 mmHg, or a cardiac index less than 2.2 L/min.m^2^, or a serum arterial lactate greater than 18 mg/dl, and pulmonary artery wedge pressure less than 15 mmHg.	18	11	5/7	3/4
Metildi *et al*. [15]	1984	N/A	N/A	N/A	N/A	N/A
The SAFE study investigators [6]	2010	Septic shock was defined by the presence of a defined focus of infection and at least two of the four systemic inflammatory response syndrome criteria and infection-related cardiovascular sequential organ failure assessment score of 3 or 4.	438	160	70/209	90/229
The CRISTAL study investigators [14]	2013	Septic shock was defined by sepsis induced acute hypovolemia. Hypovolemia was defined by the combination of (1) hypotension: systolic arterial pressure of less than 90 mm Hg, mean arterial pressure of less than 60 mm Hg, orthostatic hypotension, or a delta pulse pressure of 13% or higher; (2) evidence for low filling pressures and low cardiac index as assessed either invasively or noninvasively; and (3) signs of tissue hypoperfusion or hypoxia, including at least two of the following clinical symptoms: a Glasgow coma scale score of less than 12, mottled skin, urinary output of less than 25 mL/h, or capillary refilling time of 3 seconds or longer; and arterial lactate levels higher than 2 mmol/L, blood urea nitrogen higher than 56 mg/dL, or a fractional excretion of sodium of less than 1%.	1553	441 (28-day) 538 (90-day)	19/59 (28-day) 22/59 (90-day)	157/557 (28-day) 197/557 (90-day)
The ALBIOS study investigators [9]	2014	Septic shock was defined by the presence of a defined focus of infection and at least two of the four systemic inflammatory response syndrome criteria and infection-related cardiovascular sequential organ failure assessment score of 3 or 4.	1121	524 (90-day)	243/558 (90-day)	281/563 (90-day)

N/A, not applicable. No detailed description of hemodynamic instability in Metildi *et al*. [15] trial.

### Albumin versus saline for the outcome in patients with severe sepsis

**Figure 5:**
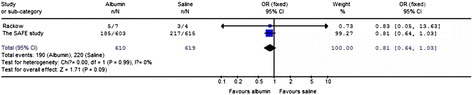
**Albumin versus saline in the resuscitation of patients with severe sepsis.**

The impact of albumin on mortality in patients with severe sepsis compared with saline was estimated from two trials [6,13] (Figure 5), the heterogeneity was determined to be non-significant (*P* = 0.99, I^2^ = 0%). Compared with saline, the use of albumin for resuscitation slightly improved the outcome in severe sepsis patients (OR 0.81; 95% CI, 0.64 to 1.03; *P* = 0.09); however, the results were not statistically significant.

## Discussion

Although previous meta-analyses [7,8,16-19] have explored the impact of albumin in resuscitating critically ill and sepsis patients, few investigators focused on the use of albumin in patients with severe sepsis, especially with septic shock. Our analysis was the only meta-analysis focusing totally on severe sepsis and septic shock, which are the most important and specific subset of sepsis, and for that the analysis separated from previous meta-analyses studies. Furthermore, to our knowledge, no meta-analysis has compared albumin with saline in severe sepsis patients up to now. In this meta-analysis, the effect of albumin as a resuscitation fluid on mortality in severe sepsis and septic shock was investigated. The results suggested a trend toward reduced 90-day mortality in severe sepsis patients resuscitated with albumin compared with crystalloid and saline. Moreover, the use of albumin for resuscitation significantly decreased the 90-day mortality in septic shock patients.

A meta-analysis is imminent for stressing the role of albumin in resuscitating patients with severe sepsis and septic shock. As the major and specific subsets of sepsis, it was reported the mortality of severe sepsis without shock was 14 to 30%, while in septic shock, the mortality rate raised to 22 to 40% [20]. Therefore, multiple randomized controlled trials have attempted to identify effective treatments to improve the survival of patients with severe sepsis and septic shock, rather than sepsis. However, the latest ALBIOS study published a disappointing result. To determine whether severe sepsis and septic shock patient benefit from the use of albumin for resuscitation compared with crystalloid, and to explore a better solution to improve the outcome, we conducted this meta-analysis to observe the effect of albumin on mortality in the resuscitation of severe sepsis and septic shock patients.

Our results were not equivalent with the recent study published by Patel *et al*. [8], which showed albumin was ineffective at reducing all-cause mortality. The differences come from: the variant target study population, the more comprehensive included studies, the distinct comparison, the dissimilar results and conclusion. As a result, our manuscript might add new evidence to help guide albumin as an optimal resuscitation fluid for septic shock.

Although the study published by Patel *et al*. did assess the role of albumin in sepsis of any severity, they did not highlight its role in severe sepsis and septic shock. As a result, the study lacked the detailed description of the characteristics of the included studies. Moreover, the data of septic shock from some important studies (for example the SAFE study) were incomplete owing to the unsuccessful data requests.

The comparison was goals-explicit in our study. As the unsolved, fundamental challenge in resuscitating severe sepsis and septic shock, it is recommended that albumin should be added to the initial resuscitation with crystalloid, and the application of hydroxyethyl starches was against given their role in renal injury [21,22]. However, there is concern about which agent would improve the outcome of the patients with severe sepsis and septic shock, especially the comparison between albumin and crystalloid. As the most commonly used crystalloid, we also compare albumin with saline.

Compared with the review published by Patel *et al*., our meta-analysis included more comprehensive studies, which might result a more accurate conclusion. There are four studies included in the *British Medical Journal* review comparing albumin with crystalloid in patients with septic shock, and half of the included studies were confronted with the ‘same author’ or ‘same data’ issue. Moreover, the data of septic shock in the well-powered SAFE study are not included.

Various reasonable explanations are available for the encouraging results using albumin in septic shock patients. Fluid expansion is a life-saving management for resuscitating septic shock patients. The goal of fluid resuscitation is to expand the intravascular space and restore effective volume. However, fluid often moves into the extravascular space and induces tissue edema in septic patients. While exploiting oncotic pressure gradients [23] to counteract the movement [24], albumin provides intravascular volume expansion more effectively and restores the fluid in the intravascular space. All these above factors affect fluid resuscitation in a more timely and efficient manner. In addition, albumin is a key transporter and binder of active molecules [25,26]. Albumin also acts as a radical-scavenging antioxidant [27], its role on inhibition of platelet aggregation [28] and maintenance of the capillary membrane permeability [29] also takes a great part in being beneficial to outcome.

This meta-analysis has some limitations that should be noted. First, only five trials were included, and two of these trials had small sample sizes. Second, some included trials were subgroups of large randomized controlled studies with variable methodological quality. Thus, the results are potentially overstated [30]. In addition, the endpoint was different in each study. Some of the trials included both 28-day and 90-day mortality values, whereas one trial only reported 28-day mortality. The remaining trials reported hospital mortality. The mortality rates obtained from various time points may influence the overall results. Finally, the concentration of albumin used in each study was different, and contained two colloid families, the hypooncotic and hyperoncotic albumin. The differences in formulation potentially do not influence the results in the same manner.

There are two other highly anticipated ongoing studies of albumin in patients with septic shock. The Five Percent Albumin vs. Normal Saline as Fluid Resuscitation Strategies for the Management of Early Suspected Septic Shock (NCT00819416) study has enrolled 47 patients up to now. The Early Albumin Resuscitation during Septic Shock (NCT00327704) study completed enrollment of 794 patients in January 2011 though it has not been updated for three years. All the results of the studies above will contribute significantly more to further guidance of the optimal resuscitation fluid for septic shock.

## Conclusions

In this meta-analysis, the results suggested a trend toward reduced 90-day mortality in severe sepsis patients resuscitated with albumin compared with crystalloid and saline. Moreover, the 90-day mortality of septic shock patients decreased significantly. Further large randomized controlled trials are necessary to determine the potential benefits of albumin in patients with severe sepsis and septic shock.

## Key messages

- Compared with crystalloid, a trend toward reduced 90-day mortality was observed in adults with severe sepsis resuscitated with albumin.
- Compared with crystalloid, the use of albumin for resuscitation significantly decreased 90-day mortality in adult septic shock patients.
- Compared with saline, the use of albumin for resuscitation slightly improved the outcome in adult severe sepsis patients.

## Additional files

Additional file 1:
**Search strategy: the file includes electronic database search strategy.**
Additional file 2:
**Excluded studies: the file includes all excluded full-text articles.**
Additional file 3:
**Characteristics of the included studies in the meta-analysis.**

## Abbreviations

ALBIOS study: the Albumin Italian Outcome Sepsis study
CI: confidence interval
OR: odds ratio
SAFE study: the Saline versus Albumin Fluid Evaluation study
