# Supplementary material for: Comparison of the effects of albumin and crystalloid on mortality in adult patients with severe sepsis and septic shock: a meta-analysis of randomized clinical trials
Source: Crit Care. 2014 Dec 15;18(6):702. doi: 10.1186/s13054-014-0702-y (PMC4284920; doi:10.1186/s13054-014-0702-y)
Supplement: Additional file 1: — Search strategy: the file includes electronic database search strategy. [file 13054_2014_702_MOESM1_ESM.doc]

**Additional File 1.** Search strategy

**Search strategy**

Database: MEDLINE via Pubmed

------------------------------------------------------------------------

## 1 (“albumin” or “serum albumin” or “albumin replacement” or “colloid” or “crystalloid” or “crystalloid solution” or “saline” or “normal saline” or “Ringer's solution” or “bicarbonated Ringer's solution” or “lactated Ringer's solution” or “isotonic solution” or “hypertonic saline”) and (“sepsis” or “severe sepsis” or “septic shock” or “critical ill” or “critical illness” or “intensive care unit” or “intensive care” or “critical care” or “ICU”) / (19,488)

## 2 limit 1 to Clinical Trial (2,385)

3 limit 2 to humans (2,319)

Database: Elsevier

--------------------------------------------------------------------------------

1 (“albumin” or “serum albumin” or “albumin replacement” or “colloid” or “crystalloid” or “crystalloid solution” or “saline” or “normal saline” or “Ringer's solution” or “bicarbonated Ringer's solution” or “lactated Ringer's solution” or “isotonic solution” or “hypertonic saline”) and (“sepsis” or “severe sepsis” or “septic shock” or “critical ill” or “critical illness” or “intensive care unit” or “intensive care” or “critical care” or “ICU”) / (386)

2 limit 1 to humans and clinical trial (293)

Database: The Cochrane Controlled Trials Register --------------------------------------------------------------------------------

1 (“albumin” or “serum albumin” or “albumin replacement” or “colloid” or “crystalloid” or “crystalloid solution” or “saline” or “normal saline” or “Ringer's solution” or “bicarbonated Ringer's solution” or “lactated Ringer's solution” or “isotonic solution” or “hypertonic saline”) and (“sepsis” or “severe sepsis” or “septic shock” or “critical ill” or “critical illness” or “intensive care unit” or “intensive care” or “critical care” or “ICU”) / (3,016)

2 limit 1 to trials (2,014)

3 limit 2 to humans (293)

Database: Web of Science (SCI/SSCI/AHCI/CPCI)

--------------------------------------------------------------------------------

1 (“albumin” or “serum albumin” or “albumin replacement” or “colloid” or “crystalloid” or “crystalloid solution” or “saline” or “normal saline” or “Ringer's solution” or “bicarbonated Ringer's solution” or “lactated ringer's solution” or “isotonic solution” or “hypertonic saline”) and (“sepsis” or “severe sepsis” or “septic shock” or “critical ill” or “critical illness” or “intensive care unit” or “intensive care” or “critical care” or “ICU”) / (14,206)

2 limit 1 to clinical trial (908)
