# Supplementary material for: Comparison of the effects of albumin and crystalloid on mortality in adult patients with severe sepsis and septic shock: a meta-analysis of randomized clinical trials
Source: Crit Care. 2014 Dec 15;18(6):702. doi: 10.1186/s13054-014-0702-y (PMC4284920; doi:10.1186/s13054-014-0702-y)
Supplement: Additional file 2: — Excluded studies: the file includes all excluded full-text articles. [file 13054_2014_702_MOESM2_ESM.doc]

**Additional File 2.** Excluded articles and reasons

**Excluded articles and reasons**

| **First author** | **Title** | **Reason for exclusion** |
| --- | --- | --- |
| Dahn 1979 [1] | Negative inotropic effect of albumin resuscitation for shock. | 1. Seriously injured patients were included in the study, data of severe sepsis was inadequate.  2. No comparison between albumin and crystalloid. |
| Lucas 1979 [2] | Impaired salt and water excretion after albumin resuscitation for hypovolemic shock. | 1. Hypovolemic shock patients were included in the study, data of severe sepsis was inadequate.  2. No comparison between albumin and crystalloid. |
| Lucas 1980 [3] | Impaired pulmonary function after albumin resuscitation from shock. | 1. Shock patients were included in the study, data of severe sepsis was inadequate.  2. No comparison between albumin and crystalloid. |
| Brown 1988 [4] | Effect of albumin supplementation during parenteral nutrition on hospital morbidity. | 1. Patients required central total parenteral nutrition were included in the study, data of severe sepsis was inadequate.  2. No comparison between albumin and crystalloid. |
| Foley 1990 [5] | Albumin supplementation in the critically ill. | 1. Hypoalbuminemic patients were included in the study, data of severe sepsis was inadequate.  2. No comparison between albumin and crystalloid. |
| Younes 1992 [6] | Hypertonic solutions in the treatment of hypovolemic shock: a prospective, randomized study in patients admitted to the emergency room. | 1. Hypovolemic shock patients were included in the study, data of severe sepsis was inadequate.  2. No comparison between albumin and crystalloid. |
| Stockwell 1992 [7] | Colloid solutions in the critically ill. A randomised comparison of albumin and polygeline. 1. Outcome and duration of stay in the intensive care unit. | 1. All patients were included in the study, data of severe sepsis was inadequate.  2. Comparison was between albumin and colloid, not between albumin and crystalloid. |
| Tuchschmidt 1992 [8] | Elevation of cardiac output and oxygen delivery improves outcome in septic shock. | 1. Septic shock patients were treated by an algorithm to increase CI to different level.  2. No comparison between albumin and crystalloid. |
| Golub 1994 [9] | Efficacy of albumin supplementation in the surgical intensive care unit: a prospective, randomized study. | 1. The study was conducted in the surgical intensive care unit, data of severe sepsis is inadequate. |
| Steltzer 1994 [10] | Haemodynamic evaluation during small volume resuscitation in patients with acute respiratory failure. | 1. Acute respiratory failure patients were included in the study, data of severe sepsis was inadequate.  2. No comparison between albumin and crystalloid. |
| Boldt 1996 [11] | The effects of albumin versus hydroxyethyl starch solution on cardiorespiratory and circulatory variables in critically ill patients. | 1. Comparison was between albumin and low-molecular weight hydroxyethyl starch solution, not between albumin and crystalloid. |
| Rock 1997 [12] | Pentastarch instead of albumin as replacement fluid for therapeutic plasma exchange. | 1. Comparison was between albumin and Pentastarch, not between albumin and crystalloid. |
| Rubin 1997 [13] | Randomized, double blind study of intravenous human albumin in hypoalbuminemic patients receiving total parenteral nutrition. | 1. Hypoalbuminemic patients were included in the study, data of severe sepsis was inadequate. |
| Ernest 1999 [14] | Distribution of normal saline and 5% albumin infusions in septic patients. | 1. Septic patients were included in the study, data of severe sepsis was inadequate. |
| Wu 2001 [15] | Hemodynamic response of modified fluid gelatin compared with lactated ringer’s solution for volume expansion in emergency resuscitation of hypovolemic shock patients: preliminary report of a prospective, randomized trial. | 1. Hypovolemic shock patients were included in the study, data of severe sepsis was inadequate.  2. Comparison was between gelatin and lactated ringer’s solution, not between albumin and crystalloid. |
| Oliveira 2002 [16] | Acute haemodynamic effects of a hypertonic saline/dextran solution in stable patients with severe sepsis. | 1. Comparison was between hypertonic saline/dextran solution and saline, not between albumin and crystalloid. |
| Quinlan 2004 [17] | Albumin influences total plasma antioxidant capacity favorably in patients with acute lung injury. | 1. Acute lung injury patients were included in the study, data of severe sepsis was inadequate. |
| Veneman 2004 [18] | Human albumin and starch administration in critically patients: a prospective randomized clinical trial. | 1. Severe sepsis patients were included in the study, mortality data of albumin group and crystalloid group was inadequate. |
| Vincent 2005 [19] | Is albumin administration in the acutely ill associated with increased mortality? Results of the SOAP study. | 1. Not randomized controlled or parallel clinical trial |
| [SAFE study investigators](http://www.ncbi.nlm.nih.gov/pubmed?term=SAFE Study Investigators%5BCorporate Author%5D) 2006 [20] | Effect of baseline serum albumin concentration on outcome of resuscitation with albumin or saline in patients in intensive care units: analysis of data from the saline versus albumin fluid evaluation (SAFE) study. | 1. Patients admitted to intensive care unit were included in the study, data of severe sepsis was inadequate. |
| Palumbo 2006 [21] | The effects of hydroxyethyl starch solution in critically ill patients. | 1. Comparison was between albumin and hydroxyethyl starch, not between albumin and crystalloid. |
| Dubois 2006 [22] | Albumin administration improves organ function in critically ill hypoalbuminemic patients: A prospective, randomized, controlled, pilot study. | 1. Hypoalbuminemic patients were included in the study, data of severe sepsis was inadequate. |
| Bellomo 2006 [23] | The effects of saline or albumin resuscitation on acid-base status and serum electrolytes. | 1. Not randomized controlled or parallel clinical trial |
| McIntyre 2007 [24] | Resuscitating patients with early severe sepsis: a Canadian multicentre observational study. | 1. Not randomized controlled or parallel clinical trial |
| Guidet 2007 [25] | The COASST study: cost-effectiveness of albumin in severe sepsis and septic shock. | 1. Not randomized controlled or parallel clinical trial |
| McIntyre 2008 [26] | Fluid resuscitation in the management of early septic shock (FINESS): a randomized controlled feasibility trial. | 1. Comparison was between pentastarch and saline, not between albumin and crystalloid. |
| Friedman 2008 [27] | Hemodynamic effects of 6% and 10% hydroxyethyl starch solutions versus 4% albumin solution in septic patients. | 1. Comparison was between hydroxyethyl starch and albumin, not between albumin and crystalloid. |
| Schortgen 2008 [28] | The risk associated with hyperoncotic colloids in patients with shock. | 1. Not randomized controlled or parallel clinical trial |
| Dolecek 2009 [29] | Therapeutic influence of 20% albumin versus 6% hydroxyethylstarch on extravascular lung water in septic patients: a randomized controlled trial. | 1. Comparison was between hydroxyethyl starch and albumin, not between albumin and crystalloid. |
| Bellomo 2009 [30] | Effects of saline or albumin resuscitation on standard coagulation tests. | 1. Not randomized controlled or parallel clinical trial |
| van der Heijden 2009 [31] | Crystalloid or colloid fluid loading and pulmonary permeability, edema, and injury in septic and nonseptic critically ill patients with hypovolemia. | 1. Hypovolemic septic patients were included in the study, mortality data of albumin group and crystalloid group was inadequate. |
| Trof 2010 [32] | Greater cardiac response of colloid than saline fluid loading in septic and non-septic critically ill patients with clinical hypovolaemia. | 1. Critically ill septic patients were included in the study, mortality data of albumin group and crystalloid group was inadequate. |
| Finfer 2010 [33] | Resuscitation fluid use in critically ill adults: an international cross-sectional study in 391 intensive care units. | 1. Not randomized controlled or parallel clinical trial |
| Zhu 2011 [34] | The study of hypertonic saline and hydroxyethyl starch treating severe sepsis. | 1. Comparison was between hypertonic saline and hydroxyethyl starch, not between albumin and crystalloid. |
| [Crystalloid versus Hydroxyethyl Starch Trial (CHEST) Management Committee](http://www.ncbi.nlm.nih.gov/pubmed?term="Crystalloid versus Hydroxyethyl Starch Trial (CHEST) Management Committee"%5BCorporate Author%5D) 2011 [35] | The crystalloid versus hydroxyethyl starch trial: protocol for a multi-centre randomised controlled trial of fluid resuscitation with 6% hydroxyl starch (130/0.4) compared to 0.9% sodium chloride (saline) in intensive care patients on mortality. | 1. Comparison was between crystalloid and hydroxyethyl starch, not between albumin and crystalloid. |
| [Scandinavian Critical Care Trials Group](http://www.ncbi.nlm.nih.gov/pubmed?term=Scandinavian Critical Care Trials Group%5BCorporate Author%5D) 2011 [36] | Comparing the effect of hydroxyethyl starch 130/0.4 with balanced crystalloid solution on mortality and kidney failure in patients with severe sepsis (6S--Scandinavian Starch for Severe Sepsis/Septic Shock trial): study protocol, design and rationale for a double-blinded, randomised clinical trial. | 1. Comparison was between crystalloid and hydroxyethyl starch, not between albumin and crystalloid. |
| McIntyre 2012 [37] | Fluid Resuscitation with 5% albumin versus Normal Saline in Early Septic Shock: a pilot randomized, controlled trial. | 1. Septic shock patients were included in the study, mortality data of albumin group and saline group was inadequate. |
| van Haren 2012 [38] | Hypertonic fluid administration in patients with septic shock: a prospective randomized controlled pilot study. | 1. Comparison was between hypertonic fluid and isotonic fluid, not between albumin and crystalloid. |
| Myburgh 2012 [39] | Hydroxyethyl starch or saline for fluid resuscitation in intensive care. | 1. Comparison was between hydroxyethyl starch and saline, not between albumin and crystalloid. |
| Yunos 2012 [40] | Association between a chloride-liberal vs chloride-restrictive intravenous fluid administration strategy and kidney injury in critically ill adults. | 1. Not randomized controlled or parallel clinical trial |
| McIntyre 2012 [41] | The PRECISE RCT: evolution of an early septic shock fluid resuscitation trial. | 1. Septic shock patients were included in the study, mortality data of albumin group and saline group was inadequate. |
| Perez 2013 [42] | Intravenous 0.9% sodium chloride therapy does not reduce length of stay of alcohol-intoxicated patients in the emergency department: A randomised controlled trial. | 1. Acute alcohol intoxication patients were included in the study, mortality data of albumin group and saline group was inadequate.  2. Comparison was not between albumin and crystalloid. |

References

1. Dahn MS, Lucas CE, Ledgerwood AM, Higgins RF: **Negative inotropic effect of albumin resuscitation for shock.** *Surgery* 1979, **86**: 235-241.
2. Lucas CE, Ledgerwood AM, Higgins RF: **Impaired salt and water excretion after albumin resuscitation for hypovolemic shock.** *Surgery* 1979, **86**: 544-549.
3. Lucas CE, Ledgerwood AM, Higgins RF, Weaver DW: **Impaired pulmonary function after albumin resuscitation from shock.** *J Trauma* 1980, **20**: 446-451.
4. Brown RO, Bradley JE, Bekemeyer WB, Luther RW: **Effect of albumin supplementation during parenteral nutrition on hospital morbidity.** *Crit Care Med* 1988, **16**: 1177-1182.
5. Foley EF, Borlase BC, Dzik WH, Bistrian BR, Benotti PN: **Albumin supplementation in the critically ill. A prospective, randomized trial.** *Arch Surg* 1990, **125**: 739-742.
6. Younes RN, Aun F, Accioly CQ, Casale LP, Szajnbok I, Birolini D: **Hypertonic solutions in the treatment of hypovolemic shock: a prospective, randomized study in patients admitted to the emergency room.** *Surgery* 1992, **111**: 380-385.
7. Stockwell MA, Soni N, Riley B: **Colloid solutions in the critically ill. A randomised comparison of albumin and polygeline. 1. Outcome and duration of stay in the intensive care unit.** *Anaesthesia* 1992, **47**: 3-6.
8. Tuchschmidt J, Fried J, Astiz M, Rackow E: **Elevation of cardiac output and oxygen delivery improves outcome in septic shock.** *Chest* 1992, **102**: 216-220.
9. Golub R, Sorrento JJ Jr, Cantu R Jr, Nierman DM, Moideen A, Stein HD: **Efficacy of albumin supplementation in the surgical intensive care unit: a prospective, randomized study.** *Crit Care Med* 1994, **22**: 613-619.
10. Steltzer H, Owen AN, Krafft P, Weinstabl C, Hammerle AF: **Haemodynamic evaluation during small volume resuscitation in patients with acute respiratory failure.** *Eur J Emerg Med* 1994, **1**: 19-26.
11. Boldt J, Heesen M, Muller M, Pabsdorf M, Hempelmann G: **The effects of albumin versus hydroxyethyl starch solution on cardiorespiratory and circulatory variables in critically ill patients.** *Anesth Analg* 1996, **83**: 254-261.
12. Rock G, Sutton DM, Freedman J, Nair RC: **Pentastarch instead of albumin as replacement fluid for therapeutic plasma exchange. The Canadian Apheresis Group.** *J Clin Apher* 1997, **12**: 165-169.
13. Rubin H, Carlson S, DeMeo M, Ganger D, Craig RM: **Randomized, double-blind study of intravenous human albumin in hypoalbuminemic patients receiving total parenteral nutrition.** *Crit Care Med* 1997, **25**: 249-252.
14. Ernest D, Belzberg AS, Dodek PM: **Distribution of normal saline and 5% albumin infusions in septic patients.** *Crit Care Med* 1999, **27**: 46-50.
15. Wu JJ, Huang MS, Tang GJ, Kao WF, Shih HC, Su CH, Lee CH: **Hemodynamic response of modified fluid gelatin compared with lactated ringer's solution for volume expansion in emergency resuscitation of hypovolemic shock patients: preliminary report of a prospective, randomized trial.** *World J Surg* 2001, **25**: 598-602.
16. Oliveira RP, Weingartner R, Ribas EO, Moraes RS, Friedman G: **Acute haemodynamic effects of a hypertonic saline/dextran solution in stable patients with severe sepsis.** *Intensive Care Med* 2002, **28**: 1574-1581.
17. Quinlan GJ, Mumby S, Martin GS, Bernard GR, Gutteridge JM, Evans TW: **Albumin influences total plasma antioxidant capacity favorably in patients with acute lung injury.** *Crit Care Med* 2004, **32**: 755-759.
18. [Veneman TF](http://www.ncbi.nlm.nih.gov/pubmed?term=Veneman TF%5BAuthor%5D&cauthor=true&cauthor_uid=15237655), [Oude Nijhuis J](http://www.ncbi.nlm.nih.gov/pubmed?term=Oude Nijhuis J%5BAuthor%5D&cauthor=true&cauthor_uid=15237655), [Woittiez AJ](http://www.ncbi.nlm.nih.gov/pubmed?term=Woittiez AJ%5BAuthor%5D&cauthor=true&cauthor_uid=15237655): **Human albumin and starch administration in critically ill patients: a prospective randomized clinical trial.** *Wien Klin Wochenschr* 2004,**116**: 305-309.
19. Vincent JL, Sakr Y, Reinhart K, Sprung CL, Gerlach H, Ranieri VM; [**Sepsis Occurrence in Acutely Ill Patients' Investigators**](http://www.ncbi.nlm.nih.gov/pubmed?term='Sepsis Occurrence in Acutely Ill Patients' Investigators%5BCorporate Author%5D)**: Is albumin administration in the acutely ill associated with increased mortality? Results of the SOAP study.** *Critical care* 2005, **9**: R745-754.
20. [SAFE Study Investigators](http://www.ncbi.nlm.nih.gov/pubmed?term=SAFE Study Investigators%5BCorporate Author%5D), [Finfer S](http://www.ncbi.nlm.nih.gov/pubmed?term=Finfer S%5BAuthor%5D&cauthor=true&cauthor_uid=17040925), [Bellomo R](http://www.ncbi.nlm.nih.gov/pubmed?term=Bellomo R%5BAuthor%5D&cauthor=true&cauthor_uid=17040925), [McEvoy S](http://www.ncbi.nlm.nih.gov/pubmed?term=McEvoy S%5BAuthor%5D&cauthor=true&cauthor_uid=17040925), [Lo SK](http://www.ncbi.nlm.nih.gov/pubmed?term=Lo SK%5BAuthor%5D&cauthor=true&cauthor_uid=17040925), [Myburgh J](http://www.ncbi.nlm.nih.gov/pubmed?term=Myburgh J%5BAuthor%5D&cauthor=true&cauthor_uid=17040925), [Neal B](http://www.ncbi.nlm.nih.gov/pubmed?term=Neal B%5BAuthor%5D&cauthor=true&cauthor_uid=17040925), [Norton R](http://www.ncbi.nlm.nih.gov/pubmed?term=Norton R%5BAuthor%5D&cauthor=true&cauthor_uid=17040925): **Effect of baseline serum albumin concentration on outcome of resuscitation with albumin or saline in patients in intensive care units: analysis of data from the saline versus albumin fluid evaluation (SAFE) study.** *BMJ* 2006, **333**: 1044.
21. Palumbo D, Servillo G, D’Amato L, Volpe ML, Capogrosso G, De Robertis E, Piazza O, Tufano R: **The effects of hydroxyethyl starch solution in critically ill patients.** *Minerva Anestesiol* 2006, **72**: 655-664.
22. Dubois MJ, Orellana-Jimenez C, Melot C, De Backer D, Berre J, Leeman M, Brimioulle S, Appoloni O, Creteur J, Vincent JL: **Albumin administration improves organ function in critically ill hypoalbuminemic patients: A prospective, randomized, controlled, pilot study.** *Crit Care Med* 2006, **34**: 2536-2540.
23. [Bellomo R](http://www.ncbi.nlm.nih.gov/pubmed?term=Bellomo R%5BAuthor%5D&cauthor=true&cauthor_uid=16971855), [Morimatsu H](http://www.ncbi.nlm.nih.gov/pubmed?term=Morimatsu H%5BAuthor%5D&cauthor=true&cauthor_uid=16971855), [French C](http://www.ncbi.nlm.nih.gov/pubmed?term=French C%5BAuthor%5D&cauthor=true&cauthor_uid=16971855), [Cole L](http://www.ncbi.nlm.nih.gov/pubmed?term=Cole L%5BAuthor%5D&cauthor=true&cauthor_uid=16971855), [Story D](http://www.ncbi.nlm.nih.gov/pubmed?term=Story D%5BAuthor%5D&cauthor=true&cauthor_uid=16971855), [Uchino S](http://www.ncbi.nlm.nih.gov/pubmed?term=Uchino S%5BAuthor%5D&cauthor=true&cauthor_uid=16971855), [Naka T](http://www.ncbi.nlm.nih.gov/pubmed?term=Naka T%5BAuthor%5D&cauthor=true&cauthor_uid=16971855); [SAFE Study Investigators](http://www.ncbi.nlm.nih.gov/pubmed?term=SAFE Study Investigators%5BCorporate Author%5D): **The effects of saline or albumin resuscitation on acid-base status and serum electrolytes.** *Crit Care Med* 2006, **34**: 2891-2897.
24. [McIntyre LA](http://www.ncbi.nlm.nih.gov/pubmed?term=McIntyre LA%5BAuthor%5D&cauthor=true&cauthor_uid=17934160), [Fergusson D](http://www.ncbi.nlm.nih.gov/pubmed?term=Fergusson D%5BAuthor%5D&cauthor=true&cauthor_uid=17934160), [Cook DJ](http://www.ncbi.nlm.nih.gov/pubmed?term=Cook DJ%5BAuthor%5D&cauthor=true&cauthor_uid=17934160), [Nair RC](http://www.ncbi.nlm.nih.gov/pubmed?term=Nair RC%5BAuthor%5D&cauthor=true&cauthor_uid=17934160), [Bell D](http://www.ncbi.nlm.nih.gov/pubmed?term=Bell D%5BAuthor%5D&cauthor=true&cauthor_uid=17934160), [Dhingra V](http://www.ncbi.nlm.nih.gov/pubmed?term=Dhingra V%5BAuthor%5D&cauthor=true&cauthor_uid=17934160), [Hutton B](http://www.ncbi.nlm.nih.gov/pubmed?term=Hutton B%5BAuthor%5D&cauthor=true&cauthor_uid=17934160), [Magder S](http://www.ncbi.nlm.nih.gov/pubmed?term=Magder S%5BAuthor%5D&cauthor=true&cauthor_uid=17934160), [Hébert PC](http://www.ncbi.nlm.nih.gov/pubmed?term=Hébert PC%5BAuthor%5D&cauthor=true&cauthor_uid=17934160); [Canadian Critical Care Trials Group](http://www.ncbi.nlm.nih.gov/pubmed?term=Canadian Critical Care Trials Group%5BCorporate Author%5D): **Resuscitating patients with early severe sepsis: a Canadian multicentre observational study.** *Can J Anaesth* 2007, **54**: 790-798.
25. Guidet B, Mosqueda GJ, Priol G, Aegerter P: **The COASST study: cost-effectiveness of albumin in severe sepsis and septic shock.** *J Crit Care* 2007, **22**: 197-203.
26. [McIntyre LA](http://www.ncbi.nlm.nih.gov/pubmed?term=McIntyre LA%5BAuthor%5D&cauthor=true&cauthor_uid=19050085), [Fergusson D](http://www.ncbi.nlm.nih.gov/pubmed?term=Fergusson D%5BAuthor%5D&cauthor=true&cauthor_uid=19050085), [Cook DJ](http://www.ncbi.nlm.nih.gov/pubmed?term=Cook DJ%5BAuthor%5D&cauthor=true&cauthor_uid=19050085), [Rankin N](http://www.ncbi.nlm.nih.gov/pubmed?term=Rankin N%5BAuthor%5D&cauthor=true&cauthor_uid=19050085), [Dhingra V](http://www.ncbi.nlm.nih.gov/pubmed?term=Dhingra V%5BAuthor%5D&cauthor=true&cauthor_uid=19050085), [Granton J](http://www.ncbi.nlm.nih.gov/pubmed?term=Granton J%5BAuthor%5D&cauthor=true&cauthor_uid=19050085), [Magder S](http://www.ncbi.nlm.nih.gov/pubmed?term=Magder S%5BAuthor%5D&cauthor=true&cauthor_uid=19050085), [Stiell I](http://www.ncbi.nlm.nih.gov/pubmed?term=Stiell I%5BAuthor%5D&cauthor=true&cauthor_uid=19050085), [Taljaard M](http://www.ncbi.nlm.nih.gov/pubmed?term=Taljaard M%5BAuthor%5D&cauthor=true&cauthor_uid=19050085), [Hebert PC](http://www.ncbi.nlm.nih.gov/pubmed?term=Hebert PC%5BAuthor%5D&cauthor=true&cauthor_uid=19050085); [Canadian Critical Care Trials Group](http://www.ncbi.nlm.nih.gov/pubmed?term=Canadian Critical Care Trials Group%5BCorporate Author%5D): **Fluid resuscitation in the management of early septic shock (FINESS): a randomized controlled feasibility trial.** *Can J Anaesth* 2008, **55**: 819-826.
27. Friedman G, Jankowski S, Shahla M, Gomez J, Vincent JL: **Hemodynamic effects of 6% and 10% hydroxyethyl starch solutions versus 4% albumin solution in septic patients.** *J Clin Anesth* 2008, **20**: 528-533.
28. Schortgen F, Girou E, Deye N, Brochard L; [CRYCO Study Group](http://www.ncbi.nlm.nih.gov/pubmed?term=CRYCO Study Group%5BCorporate Author%5D): **The risk associated with hyperoncotic colloids in patients with shock.** *Intensive Care Med* 2008, **34**: 2157-2168.
29. [Dolecek M](http://www.ncbi.nlm.nih.gov/pubmed?term=Dolecek M%5BAuthor%5D&cauthor=true&cauthor_uid=20214205), [Svoboda P](http://www.ncbi.nlm.nih.gov/pubmed?term=Svoboda P%5BAuthor%5D&cauthor=true&cauthor_uid=20214205), [Kantorová I](http://www.ncbi.nlm.nih.gov/pubmed?term=Kantorová I%5BAuthor%5D&cauthor=true&cauthor_uid=20214205), [Scheer P](http://www.ncbi.nlm.nih.gov/pubmed?term=Scheer P%5BAuthor%5D&cauthor=true&cauthor_uid=20214205), [Sas I](http://www.ncbi.nlm.nih.gov/pubmed?term=Sas I%5BAuthor%5D&cauthor=true&cauthor_uid=20214205), [Bíbrová J](http://www.ncbi.nlm.nih.gov/pubmed?term=Bíbrová J%5BAuthor%5D&cauthor=true&cauthor_uid=20214205), [Radvanova J](http://www.ncbi.nlm.nih.gov/pubmed?term=Radvanova J%5BAuthor%5D&cauthor=true&cauthor_uid=20214205), [Radvan M](http://www.ncbi.nlm.nih.gov/pubmed?term=Radvan M%5BAuthor%5D&cauthor=true&cauthor_uid=20214205): **Therapeutic influence of 20% albumin versus 6% hydroxyethylstarch on extravascular lung water in septic patients: a randomized controlled trial.** *Hepatogastroenterology* 2009, **56**: 1622-1628.
30. [van der Heijden M](http://www.ncbi.nlm.nih.gov/pubmed?term=van der Heijden M%5BAuthor%5D&cauthor=true&cauthor_uid=19242338), [Verheij J](http://www.ncbi.nlm.nih.gov/pubmed?term=Verheij J%5BAuthor%5D&cauthor=true&cauthor_uid=19242338), [van Nieuw Amerongen GP](http://www.ncbi.nlm.nih.gov/pubmed?term=van Nieuw Amerongen GP%5BAuthor%5D&cauthor=true&cauthor_uid=19242338), [Groeneveld AB](http://www.ncbi.nlm.nih.gov/pubmed?term=Groeneveld AB%5BAuthor%5D&cauthor=true&cauthor_uid=19242338): **Crystalloid or colloid fluid loading and pulmonary permeability, edema, and injury in septic and nonseptic critically ill patients with hypovolemia.** [*Crit Care Med*](http://www.ncbi.nlm.nih.gov/pubmed/19242338) 2009, **37**: 1275-1281.
31. [Bellomo R](http://www.ncbi.nlm.nih.gov/pubmed?term=Bellomo R%5BAuthor%5D&cauthor=true&cauthor_uid=20001872), [Morimatsu H](http://www.ncbi.nlm.nih.gov/pubmed?term=Morimatsu H%5BAuthor%5D&cauthor=true&cauthor_uid=20001872), [Presneill J](http://www.ncbi.nlm.nih.gov/pubmed?term=Presneill J%5BAuthor%5D&cauthor=true&cauthor_uid=20001872), [French C](http://www.ncbi.nlm.nih.gov/pubmed?term=French C%5BAuthor%5D&cauthor=true&cauthor_uid=20001872), [Cole L](http://www.ncbi.nlm.nih.gov/pubmed?term=Cole L%5BAuthor%5D&cauthor=true&cauthor_uid=20001872), [Story D](http://www.ncbi.nlm.nih.gov/pubmed?term=Story D%5BAuthor%5D&cauthor=true&cauthor_uid=20001872), [Uchino S](http://www.ncbi.nlm.nih.gov/pubmed?term=Uchino S%5BAuthor%5D&cauthor=true&cauthor_uid=20001872), [Naka T](http://www.ncbi.nlm.nih.gov/pubmed?term=Naka T%5BAuthor%5D&cauthor=true&cauthor_uid=20001872), [Finfer S](http://www.ncbi.nlm.nih.gov/pubmed?term=Finfer S%5BAuthor%5D&cauthor=true&cauthor_uid=20001872), [Cooper DJ](http://www.ncbi.nlm.nih.gov/pubmed?term=Cooper DJ%5BAuthor%5D&cauthor=true&cauthor_uid=20001872), [Myburgh J](http://www.ncbi.nlm.nih.gov/pubmed?term=Myburgh J%5BAuthor%5D&cauthor=true&cauthor_uid=20001872); [SAFE Study Investigators and the Australian and New Zealand Intensive Care Society Clinical Trials Group](http://www.ncbi.nlm.nih.gov/pubmed?term=SAFE Study Investigators and the Australian and New Zealand Intensive Care Society Clinical Trials Group%5BCorporate Author%5D): **Effects of saline or albumin resuscitation on standard coagulation tests.** *Crit Care Resusc* 2009, **11**: 250-256.
32. [Trof RJ](http://www.ncbi.nlm.nih.gov/pubmed?term=Trof RJ%5BAuthor%5D&cauthor=true&cauthor_uid=20165941), [Sukul SP](http://www.ncbi.nlm.nih.gov/pubmed?term=Sukul SP%5BAuthor%5D&cauthor=true&cauthor_uid=20165941), [Twisk JW](http://www.ncbi.nlm.nih.gov/pubmed?term=Twisk JW%5BAuthor%5D&cauthor=true&cauthor_uid=20165941), [Girbes AR](http://www.ncbi.nlm.nih.gov/pubmed?term=Girbes AR%5BAuthor%5D&cauthor=true&cauthor_uid=20165941), [Groeneveld AB](http://www.ncbi.nlm.nih.gov/pubmed?term=Groeneveld AB%5BAuthor%5D&cauthor=true&cauthor_uid=20165941): **Greater cardiac response of colloid than saline fluid loading in septic and non-septic critically ill patients with clinical hypovolaemia.** [*Intensive Care Med*](http://www.ncbi.nlm.nih.gov/pubmed/?term=Greater+cardiac+response+of+colloid+than+saline+fluid+loading+in+septic+and+non-septic+critically+ill+patients+with+clinical+hypovolaemia) 2010, **36**: 697-701.
33. [Finfer S](http://www.ncbi.nlm.nih.gov/pubmed?term=Finfer S%5BAuthor%5D&cauthor=true&cauthor_uid=20950434), [Liu B](http://www.ncbi.nlm.nih.gov/pubmed?term=Liu B%5BAuthor%5D&cauthor=true&cauthor_uid=20950434), [Taylor C](http://www.ncbi.nlm.nih.gov/pubmed?term=Taylor C%5BAuthor%5D&cauthor=true&cauthor_uid=20950434), [Bellomo R](http://www.ncbi.nlm.nih.gov/pubmed?term=Bellomo R%5BAuthor%5D&cauthor=true&cauthor_uid=20950434), [Billot L](http://www.ncbi.nlm.nih.gov/pubmed?term=Billot L%5BAuthor%5D&cauthor=true&cauthor_uid=20950434), [Cook D](http://www.ncbi.nlm.nih.gov/pubmed?term=Cook D%5BAuthor%5D&cauthor=true&cauthor_uid=20950434), [Du B](http://www.ncbi.nlm.nih.gov/pubmed?term=Du B%5BAuthor%5D&cauthor=true&cauthor_uid=20950434), [McArthur C](http://www.ncbi.nlm.nih.gov/pubmed?term=McArthur C%5BAuthor%5D&cauthor=true&cauthor_uid=20950434), [Myburgh J](http://www.ncbi.nlm.nih.gov/pubmed?term=Myburgh J%5BAuthor%5D&cauthor=true&cauthor_uid=20950434); [SAFE TRIPS Investigators](http://www.ncbi.nlm.nih.gov/pubmed?term=SAFE TRIPS Investigators%5BCorporate Author%5D): **Resuscitation fluid use in critically ill adults: an international cross-sectional study in 391 intensive care units.** *Critical care* 2010, **14**: R185.
34. [Zhu GC](http://www.ncbi.nlm.nih.gov/pubmed?term=Zhu GC%5BAuthor%5D&cauthor=true&cauthor_uid=21366943), [Quan ZY](http://www.ncbi.nlm.nih.gov/pubmed?term=Quan ZY%5BAuthor%5D&cauthor=true&cauthor_uid=21366943), [Shao YS](http://www.ncbi.nlm.nih.gov/pubmed?term=Shao YS%5BAuthor%5D&cauthor=true&cauthor_uid=21366943), [Zhao JG](http://www.ncbi.nlm.nih.gov/pubmed?term=Zhao JG%5BAuthor%5D&cauthor=true&cauthor_uid=21366943), [Zhang YT](http://www.ncbi.nlm.nih.gov/pubmed?term=Zhang YT%5BAuthor%5D&cauthor=true&cauthor_uid=21366943): **The study of hypertonic saline and hydroxyethyl starch treating severe sepsis.** *Zhongguo wei zhong bing ji jiu yi xue* 2011, **23**: 150-153.
35. [Crystalloid versus Hydroxyethyl Starch Trial (CHEST) Management Committee](http://www.ncbi.nlm.nih.gov/pubmed?term="Crystalloid versus Hydroxyethyl Starch Trial (CHEST) Management Committee"%5BCorporate Author%5D): **The crystalloid versus hydroxyethyl starch trial: protocol for a multi-centre randomised controlled trial of fluid resuscitation with 6% hydroxyl starch (130/0.4) compared to 0.9% sodium chloride (saline) in intensive care patients on mortality.** *Intensive Care Medicine* 2011, **37**: 816-823.
36. [Perner A](http://www.ncbi.nlm.nih.gov/pubmed?term=Perner A%5BAuthor%5D&cauthor=true&cauthor_uid=21269526), [Haase N](http://www.ncbi.nlm.nih.gov/pubmed?term=Haase N%5BAuthor%5D&cauthor=true&cauthor_uid=21269526), [Wetterslev J](http://www.ncbi.nlm.nih.gov/pubmed?term=Wetterslev J%5BAuthor%5D&cauthor=true&cauthor_uid=21269526), [Aneman A](http://www.ncbi.nlm.nih.gov/pubmed?term=Aneman A%5BAuthor%5D&cauthor=true&cauthor_uid=21269526), [Tenhunen J](http://www.ncbi.nlm.nih.gov/pubmed?term=Tenhunen J%5BAuthor%5D&cauthor=true&cauthor_uid=21269526), [Guttormsen AB](http://www.ncbi.nlm.nih.gov/pubmed?term=Guttormsen AB%5BAuthor%5D&cauthor=true&cauthor_uid=21269526), [Klemenzson G](http://www.ncbi.nlm.nih.gov/pubmed?term=Klemenzson G%5BAuthor%5D&cauthor=true&cauthor_uid=21269526), [Pott F](http://www.ncbi.nlm.nih.gov/pubmed?term=Pott F%5BAuthor%5D&cauthor=true&cauthor_uid=21269526), [Bødker KD](http://www.ncbi.nlm.nih.gov/pubmed?term=Bødker KD%5BAuthor%5D&cauthor=true&cauthor_uid=21269526), [Bådstøløkken PM](http://www.ncbi.nlm.nih.gov/pubmed?term=Bådstøløkken PM%5BAuthor%5D&cauthor=true&cauthor_uid=21269526), [Bendtsen A](http://www.ncbi.nlm.nih.gov/pubmed?term=Bendtsen A%5BAuthor%5D&cauthor=true&cauthor_uid=21269526), [Søe-Jensen P](http://www.ncbi.nlm.nih.gov/pubmed?term=Søe-Jensen P%5BAuthor%5D&cauthor=true&cauthor_uid=21269526),[Tousi H](http://www.ncbi.nlm.nih.gov/pubmed?term=Tousi H%5BAuthor%5D&cauthor=true&cauthor_uid=21269526), [Bestle M](http://www.ncbi.nlm.nih.gov/pubmed?term=Bestle M%5BAuthor%5D&cauthor=true&cauthor_uid=21269526), [Pawlowicz M](http://www.ncbi.nlm.nih.gov/pubmed?term=Pawlowicz M%5BAuthor%5D&cauthor=true&cauthor_uid=21269526), [Winding R](http://www.ncbi.nlm.nih.gov/pubmed?term=Winding R%5BAuthor%5D&cauthor=true&cauthor_uid=21269526), [Bülow HH](http://www.ncbi.nlm.nih.gov/pubmed?term=Bülow HH%5BAuthor%5D&cauthor=true&cauthor_uid=21269526), [Kancir C](http://www.ncbi.nlm.nih.gov/pubmed?term=Kancir C%5BAuthor%5D&cauthor=true&cauthor_uid=21269526), [Steensen M](http://www.ncbi.nlm.nih.gov/pubmed?term=Steensen M%5BAuthor%5D&cauthor=true&cauthor_uid=21269526), [Nielsen J](http://www.ncbi.nlm.nih.gov/pubmed?term=Nielsen J%5BAuthor%5D&cauthor=true&cauthor_uid=21269526), [Fogh B](http://www.ncbi.nlm.nih.gov/pubmed?term=Fogh B%5BAuthor%5D&cauthor=true&cauthor_uid=21269526), [Madsen KR](http://www.ncbi.nlm.nih.gov/pubmed?term=Madsen KR%5BAuthor%5D&cauthor=true&cauthor_uid=21269526), [Larsen NH](http://www.ncbi.nlm.nih.gov/pubmed?term=Larsen NH%5BAuthor%5D&cauthor=true&cauthor_uid=21269526), [Carlsson M](http://www.ncbi.nlm.nih.gov/pubmed?term=Carlsson M%5BAuthor%5D&cauthor=true&cauthor_uid=21269526), [Wiis J](http://www.ncbi.nlm.nih.gov/pubmed?term=Wiis J%5BAuthor%5D&cauthor=true&cauthor_uid=21269526), [Petersen JA](http://www.ncbi.nlm.nih.gov/pubmed?term=Petersen JA%5BAuthor%5D&cauthor=true&cauthor_uid=21269526),[Iversen S](http://www.ncbi.nlm.nih.gov/pubmed?term=Iversen S%5BAuthor%5D&cauthor=true&cauthor_uid=21269526), [Schøidt O](http://www.ncbi.nlm.nih.gov/pubmed?term=Schøidt O%5BAuthor%5D&cauthor=true&cauthor_uid=21269526), [Leivdal S](http://www.ncbi.nlm.nih.gov/pubmed?term=Leivdal S%5BAuthor%5D&cauthor=true&cauthor_uid=21269526), [Berezowicz P](http://www.ncbi.nlm.nih.gov/pubmed?term=Berezowicz P%5BAuthor%5D&cauthor=true&cauthor_uid=21269526), [Pettilä V](http://www.ncbi.nlm.nih.gov/pubmed?term=Pettilä V%5BAuthor%5D&cauthor=true&cauthor_uid=21269526), [Ruokonen E](http://www.ncbi.nlm.nih.gov/pubmed?term=Ruokonen E%5BAuthor%5D&cauthor=true&cauthor_uid=21269526), [Klepstad P](http://www.ncbi.nlm.nih.gov/pubmed?term=Klepstad P%5BAuthor%5D&cauthor=true&cauthor_uid=21269526), [Karlsson S](http://www.ncbi.nlm.nih.gov/pubmed?term=Karlsson S%5BAuthor%5D&cauthor=true&cauthor_uid=21269526), [Kaukonen M](http://www.ncbi.nlm.nih.gov/pubmed?term=Kaukonen M%5BAuthor%5D&cauthor=true&cauthor_uid=21269526), [Rutanen J](http://www.ncbi.nlm.nih.gov/pubmed?term=Rutanen J%5BAuthor%5D&cauthor=true&cauthor_uid=21269526), [Karason S](http://www.ncbi.nlm.nih.gov/pubmed?term=Karason S%5BAuthor%5D&cauthor=true&cauthor_uid=21269526), [Kjældgaard AL](http://www.ncbi.nlm.nih.gov/pubmed?term=Kjældgaard AL%5BAuthor%5D&cauthor=true&cauthor_uid=21269526), [Holst LB](http://www.ncbi.nlm.nih.gov/pubmed?term=Holst LB%5BAuthor%5D&cauthor=true&cauthor_uid=21269526),[Wernerman J](http://www.ncbi.nlm.nih.gov/pubmed?term=Wernerman J%5BAuthor%5D&cauthor=true&cauthor_uid=21269526); [Scandinavian Critical Care Trials Group](http://www.ncbi.nlm.nih.gov/pubmed?term=Scandinavian Critical Care Trials Group%5BCorporate Author%5D): **Comparing the effect of hydroxyethyl starch 130/0.4 with balanced crystalloid solution on mortality and kidney failure in patients with severe sepsis (6S--Scandinavian Starch for Severe Sepsis/Septic Shock trial): study protocol, design and rationale for a double-blinded, randomised clinical trial.** [*Trials*](http://www.ncbi.nlm.nih.gov/pubmed/21269526) 2011, **12**:24.
37. [McIntyre LA](http://www.ncbi.nlm.nih.gov/pubmed?term=McIntyre LA%5BAuthor%5D&cauthor=true&cauthor_uid=22176806), [Fergusson DA](http://www.ncbi.nlm.nih.gov/pubmed?term=Fergusson DA%5BAuthor%5D&cauthor=true&cauthor_uid=22176806), [Cook DJ](http://www.ncbi.nlm.nih.gov/pubmed?term=Cook DJ%5BAuthor%5D&cauthor=true&cauthor_uid=22176806), [Rowe BH](http://www.ncbi.nlm.nih.gov/pubmed?term=Rowe BH%5BAuthor%5D&cauthor=true&cauthor_uid=22176806), [Bagshaw SM](http://www.ncbi.nlm.nih.gov/pubmed?term=Bagshaw SM%5BAuthor%5D&cauthor=true&cauthor_uid=22176806), [Easton D](http://www.ncbi.nlm.nih.gov/pubmed?term=Easton D%5BAuthor%5D&cauthor=true&cauthor_uid=22176806), [Emond M](http://www.ncbi.nlm.nih.gov/pubmed?term=Emond M%5BAuthor%5D&cauthor=true&cauthor_uid=22176806), [Finfer S](http://www.ncbi.nlm.nih.gov/pubmed?term=Finfer S%5BAuthor%5D&cauthor=true&cauthor_uid=22176806), [Fox-Robichaud A](http://www.ncbi.nlm.nih.gov/pubmed?term=Fox-Robichaud A%5BAuthor%5D&cauthor=true&cauthor_uid=22176806), [Gaudert C](http://www.ncbi.nlm.nih.gov/pubmed?term=Gaudert C%5BAuthor%5D&cauthor=true&cauthor_uid=22176806), [Green R](http://www.ncbi.nlm.nih.gov/pubmed?term=Green R%5BAuthor%5D&cauthor=true&cauthor_uid=22176806), [Hebert P](http://www.ncbi.nlm.nih.gov/pubmed?term=Hebert P%5BAuthor%5D&cauthor=true&cauthor_uid=22176806), [Marshall J](http://www.ncbi.nlm.nih.gov/pubmed?term=Marshall J%5BAuthor%5D&cauthor=true&cauthor_uid=22176806),[Rankin N](http://www.ncbi.nlm.nih.gov/pubmed?term=Rankin N%5BAuthor%5D&cauthor=true&cauthor_uid=22176806), [Stiell I](http://www.ncbi.nlm.nih.gov/pubmed?term=Stiell I%5BAuthor%5D&cauthor=true&cauthor_uid=22176806), [Tinmouth A](http://www.ncbi.nlm.nih.gov/pubmed?term=Tinmouth A%5BAuthor%5D&cauthor=true&cauthor_uid=22176806), [Pagliarello J](http://www.ncbi.nlm.nih.gov/pubmed?term=Pagliarello J%5BAuthor%5D&cauthor=true&cauthor_uid=22176806), [Turgeon AF](http://www.ncbi.nlm.nih.gov/pubmed?term=Turgeon AF%5BAuthor%5D&cauthor=true&cauthor_uid=22176806), [Worster A](http://www.ncbi.nlm.nih.gov/pubmed?term=Worster A%5BAuthor%5D&cauthor=true&cauthor_uid=22176806), [Zarychanski R](http://www.ncbi.nlm.nih.gov/pubmed?term=Zarychanski R%5BAuthor%5D&cauthor=true&cauthor_uid=22176806); [Canadian Critical Care Trials Group](http://www.ncbi.nlm.nih.gov/pubmed?term=Canadian Critical Care Trials Group%5BCorporate Author%5D): **Fluid Resuscitation with 5% albumin versus Normal Saline in Early Septic Shock: a pilot randomized, controlled trial.** *J Crit Care* 2012, **27**: 317.e1-6.
38. [van Haren FM](http://www.ncbi.nlm.nih.gov/pubmed?term=van Haren FM%5BAuthor%5D&cauthor=true&cauthor_uid=22089205), [Sleigh J](http://www.ncbi.nlm.nih.gov/pubmed?term=Sleigh J%5BAuthor%5D&cauthor=true&cauthor_uid=22089205), [Boerma EC](http://www.ncbi.nlm.nih.gov/pubmed?term=Boerma EC%5BAuthor%5D&cauthor=true&cauthor_uid=22089205), [La Pine M](http://www.ncbi.nlm.nih.gov/pubmed?term=La Pine M%5BAuthor%5D&cauthor=true&cauthor_uid=22089205), [Bahr M](http://www.ncbi.nlm.nih.gov/pubmed?term=Bahr M%5BAuthor%5D&cauthor=true&cauthor_uid=22089205), [Pickkers P](http://www.ncbi.nlm.nih.gov/pubmed?term=Pickkers P%5BAuthor%5D&cauthor=true&cauthor_uid=22089205), [van der Hoeven JG](http://www.ncbi.nlm.nih.gov/pubmed?term=van der Hoeven JG%5BAuthor%5D&cauthor=true&cauthor_uid=22089205): **Hypertonic fluid administration in patients with septic shock: a prospective randomized controlled pilot study.** *Shock* 2012, **37**: 268-275.
39. [Myburgh JA](http://www.ncbi.nlm.nih.gov/pubmed?term=Myburgh JA%5BAuthor%5D&cauthor=true&cauthor_uid=23075127), [Finfer S](http://www.ncbi.nlm.nih.gov/pubmed?term=Finfer S%5BAuthor%5D&cauthor=true&cauthor_uid=23075127), [Bellomo R](http://www.ncbi.nlm.nih.gov/pubmed?term=Bellomo R%5BAuthor%5D&cauthor=true&cauthor_uid=23075127), [Billot L](http://www.ncbi.nlm.nih.gov/pubmed?term=Billot L%5BAuthor%5D&cauthor=true&cauthor_uid=23075127), [Cass A](http://www.ncbi.nlm.nih.gov/pubmed?term=Cass A%5BAuthor%5D&cauthor=true&cauthor_uid=23075127), [Gattas D](http://www.ncbi.nlm.nih.gov/pubmed?term=Gattas D%5BAuthor%5D&cauthor=true&cauthor_uid=23075127), [Glass P](http://www.ncbi.nlm.nih.gov/pubmed?term=Glass P%5BAuthor%5D&cauthor=true&cauthor_uid=23075127), [Lipman J](http://www.ncbi.nlm.nih.gov/pubmed?term=Lipman J%5BAuthor%5D&cauthor=true&cauthor_uid=23075127), [Liu B](http://www.ncbi.nlm.nih.gov/pubmed?term=Liu B%5BAuthor%5D&cauthor=true&cauthor_uid=23075127), [McArthur C](http://www.ncbi.nlm.nih.gov/pubmed?term=McArthur C%5BAuthor%5D&cauthor=true&cauthor_uid=23075127), [McGuinness S](http://www.ncbi.nlm.nih.gov/pubmed?term=McGuinness S%5BAuthor%5D&cauthor=true&cauthor_uid=23075127), [Rajbhandari D](http://www.ncbi.nlm.nih.gov/pubmed?term=Rajbhandari D%5BAuthor%5D&cauthor=true&cauthor_uid=23075127), [Taylor CB](http://www.ncbi.nlm.nih.gov/pubmed?term=Taylor CB%5BAuthor%5D&cauthor=true&cauthor_uid=23075127), [Webb SA](http://www.ncbi.nlm.nih.gov/pubmed?term=Webb SA%5BAuthor%5D&cauthor=true&cauthor_uid=23075127); [CHEST Investigators](http://www.ncbi.nlm.nih.gov/pubmed?term=CHEST Investigators%5BCorporate Author%5D); [Australian and New Zealand Intensive Care Society Clinical Trials Group](http://www.ncbi.nlm.nih.gov/pubmed?term=Australian and New Zealand Intensive Care Society Clinical Trials Group%5BCorporate Author%5D): **Hydroxyethyl starch or saline for fluid resuscitation in intensive care.** *N Engl J Med* 2012, **367**: 1901-1911.
40. Yunos NM, Bellomo R, Hegarty C, Story D, Ho L, Bailey M: **Association between a chloride-liberal vs chloride-restrictive intravenous fluid administration strategy and kidney injury in critically ill adults.** *JAMA* 2012, **308**: 1566-1572.
41. [McIntyre L](http://www.ncbi.nlm.nih.gov/pubmed?term=McIntyre L%5BAuthor%5D&cauthor=true&cauthor_uid=22222146), [Fergusson DA](http://www.ncbi.nlm.nih.gov/pubmed?term=Fergusson DA%5BAuthor%5D&cauthor=true&cauthor_uid=22222146), [Rowe B](http://www.ncbi.nlm.nih.gov/pubmed?term=Rowe B%5BAuthor%5D&cauthor=true&cauthor_uid=22222146), [Cook DJ](http://www.ncbi.nlm.nih.gov/pubmed?term=Cook DJ%5BAuthor%5D&cauthor=true&cauthor_uid=22222146), [Arabi Y](http://www.ncbi.nlm.nih.gov/pubmed?term=Arabi Y%5BAuthor%5D&cauthor=true&cauthor_uid=22222146), [Bagshaw SM](http://www.ncbi.nlm.nih.gov/pubmed?term=Bagshaw SM%5BAuthor%5D&cauthor=true&cauthor_uid=22222146), [Emond M](http://www.ncbi.nlm.nih.gov/pubmed?term=Emond M%5BAuthor%5D&cauthor=true&cauthor_uid=22222146), [Finfer S](http://www.ncbi.nlm.nih.gov/pubmed?term=Finfer S%5BAuthor%5D&cauthor=true&cauthor_uid=22222146), [Fox-Robichaud A](http://www.ncbi.nlm.nih.gov/pubmed?term=Fox-Robichaud A%5BAuthor%5D&cauthor=true&cauthor_uid=22222146), [Gray A](http://www.ncbi.nlm.nih.gov/pubmed?term=Gray A%5BAuthor%5D&cauthor=true&cauthor_uid=22222146), [Green R](http://www.ncbi.nlm.nih.gov/pubmed?term=Green R%5BAuthor%5D&cauthor=true&cauthor_uid=22222146), [Hebert P](http://www.ncbi.nlm.nih.gov/pubmed?term=Hebert P%5BAuthor%5D&cauthor=true&cauthor_uid=22222146), [Lang E](http://www.ncbi.nlm.nih.gov/pubmed?term=Lang E%5BAuthor%5D&cauthor=true&cauthor_uid=22222146), [Marshall J](http://www.ncbi.nlm.nih.gov/pubmed?term=Marshall J%5BAuthor%5D&cauthor=true&cauthor_uid=22222146), [Stiell I](http://www.ncbi.nlm.nih.gov/pubmed?term=Stiell I%5BAuthor%5D&cauthor=true&cauthor_uid=22222146), [Tinmouth A](http://www.ncbi.nlm.nih.gov/pubmed?term=Tinmouth A%5BAuthor%5D&cauthor=true&cauthor_uid=22222146), [Pagliarello J](http://www.ncbi.nlm.nih.gov/pubmed?term=Pagliarello J%5BAuthor%5D&cauthor=true&cauthor_uid=22222146), [Turgeon A](http://www.ncbi.nlm.nih.gov/pubmed?term=Turgeon A%5BAuthor%5D&cauthor=true&cauthor_uid=22222146), [Walsh T](http://www.ncbi.nlm.nih.gov/pubmed?term=Walsh T%5BAuthor%5D&cauthor=true&cauthor_uid=22222146), [Worster A](http://www.ncbi.nlm.nih.gov/pubmed?term=Worster A%5BAuthor%5D&cauthor=true&cauthor_uid=22222146), [Zarychanski R](http://www.ncbi.nlm.nih.gov/pubmed?term=Zarychanski R%5BAuthor%5D&cauthor=true&cauthor_uid=22222146); [Canadian Critical Care Trials Group](http://www.ncbi.nlm.nih.gov/pubmed?term=Canadian Critical Care Trials Group%5BCorporate Author%5D): **The PRECISE RCT: evolution of an early septic shock fluid resuscitation trial.** *Transfus Med Rev* 2012, **26**: 333-341.
42. [Perez SR](http://www.ncbi.nlm.nih.gov/pubmed?term=Perez SR%5BAuthor%5D&cauthor=true&cauthor_uid=24308613), [Keijzers G](http://www.ncbi.nlm.nih.gov/pubmed?term=Keijzers G%5BAuthor%5D&cauthor=true&cauthor_uid=24308613), [Steele M](http://www.ncbi.nlm.nih.gov/pubmed?term=Steele M%5BAuthor%5D&cauthor=true&cauthor_uid=24308613), [Byrnes J](http://www.ncbi.nlm.nih.gov/pubmed?term=Byrnes J%5BAuthor%5D&cauthor=true&cauthor_uid=24308613), [Scuffham PA](http://www.ncbi.nlm.nih.gov/pubmed?term=Scuffham PA%5BAuthor%5D&cauthor=true&cauthor_uid=24308613): **Intravenous 0.9% sodium chloride therapy does not reduce length of stay of alcohol-intoxicated patients in the emergency department: A randomised controlled trial.** *Emerg Med Australas* 2013, **25**: 527-534.
