# Supplementary material for: Comparison of the effects of albumin and crystalloid on mortality in adult patients with severe sepsis and septic shock: a meta-analysis of randomized clinical trials
Source: Crit Care. 2014 Dec 15;18(6):702. doi: 10.1186/s13054-014-0702-y (PMC4284920; doi:10.1186/s13054-014-0702-y)
Supplement: Additional file 3: — Characteristics of the included studies in the meta-analysis. [file 13054_2014_702_MOESM3_ESM.doc]

**Table 1.** Characteristics of the included studies

| Authors | Year | Jadad scale | Study population | Total sample size | Sample size of severe sepsis | Albumin group | Crystalloid group | Intervention | Resuscitation end point | Outcome | Non-survival of severe sepsis | Non-survival of albumin group with severe sepsis (events/total) | Non-survival of crystalloid group with severe sepsis (events/total) |
| --- | --- | --- | --- | --- | --- | --- | --- | --- | --- | --- | --- | --- | --- |
| Rackow  et al ^[12]^ | 1983 | 2 | Hypovolemic circulatory shock patients | 26 | 18 | 5% human serum albumin (Cutter) | 0.9% saline, 6% hetastarch | 250 ml every 15 minutes | Pulmonary artery wedge pressure reach 15 mm Hg | Hospital mortality | 11 | 5/7 | 3/4 |
| Metildi  et al ^[14]^ | 1984 | 3 | Patients with severe pulmonary insufficiency | 46 | 24 | 5% albumin (50 g of salt-poor serum albumin in 1000 ml of Ringer’s lactate) | Ringer’s lactate | 100-250 ml as a bonus, at least 2000 ml during the study period | Maintain pH, base deficit and SvO_2_ | Hospital mortality | 21 | 10/12 | 11/12 |
| The SAFE study investigators ^[6]^ | 2010 | 5 | Patients  were  judged to require fluid administration | 6,997 | 1,218 | 4% albumin (Albumex, CSL) | 0.9% saline | Fluid resuscitation in the ICU until death, discharge  or 28 days after randomization | Decided by the treating physician | 28-day mortality | 402 | 185/603 | 217/615 |
| The CRISTAL study investigators ^[13]^ | 2013 | N/A | Adult patients require fluid resuscitation for acute hypovolemia | 2,857 | 1,553 | 4%, 5%, 20% or 25% albumin, dextrans, hydroxyethyl starches, gelatins | Isotonic or hypertonic saline and buffered solutions | Fluid resuscitation decided by the investigators | Decided by the investigators with restrictions | 28-day mortality, 90-day mortality | 441 (28-day) 538 (90-day) | 19/59 (28-day) 22/59 (90-day) | 157/557 (28-day) 197/557 (90-day) |
| The ALBIOS study investigators ^[8]^ | 2014 | 3 | Adult patients with severe sepsis | 1,818 | 1,818 | 20% albumin and crystalloid | Crystalloid | 300 ml albumin and crystalloids or crystalloid alone infusion in 3 hours followed by the study protocol | To achieve the goal of early goal directed therapy | 28-day mortality, 90-day mortality | 573 (28-day) 754 (90-day) | 285/895 (28-day)  365/888 (90-day) | 288/900 (28-day)  389/893 (90-day) |

N/A, not applicable; ICU, intensive care unit.
